# Reverse Total Shoulder Arthroplasty in a 25-Year-Old Woman With Seropositive Rheumatoid Arthritis After Prior Age-Based Surgical Deferral

**DOI:** 10.7759/cureus.116073

**Published:** 2026-09-10

**Authors:** Natalie Tripplet, Giovanni Mella-Velazquez, Timothy Griffith

**Affiliations:** 1 Rheumatology, Morehouse School of Medicine, Atlanta, USA; 2 Anesthesiology, Emory University, Atlanta, USA; 3 Orthopedics, Peachtree Orthopaedic Clinic, Atlanta, USA

**Keywords:** case report, inflammatory shoulder arthropathy, reverse total shoulder arthroplasty (rtsa), rheumatoid arthritis, rotator cuff insufficiency, young adult

## Abstract

Reverse total shoulder arthroplasty (RTSA) in very young adults with rheumatoid arthritis (RA) is uncommon because of concerns regarding implant longevity and lifetime revision burden, and outcomes data in patients younger than 30 years remain limited. We report the case of a 25-year-old woman with seropositive RA who developed rapid, destructive monoarticular involvement of the right shoulder despite treatment with disease-modifying antirheumatic drugs (DMARDs) and biologic therapy. Imaging demonstrated complete glenohumeral joint space obliteration, humeral head collapse, erosive changes, and rotator cuff insufficiency. An initial shoulder surgeon declined arthroplasty based on her age and anticipated revision risk. Following staged diagnostic arthroscopy, definitive RTSA was performed in December 2023 without complication. At four months, near-full active forward elevation was restored. At two-year follow-up, the American Shoulder and Elbow Surgeons (ASES) score was 80/100 (pain 3/10; activities of daily living 27/30), and the Single Assessment Numeric Evaluation (SANE) score was 95%. No instability, infection, or mechanical failure occurred. This case demonstrates that RTSA can provide excellent early and mid-term functional outcomes in carefully selected very young patients with end-stage inflammatory shoulder disease and may help inform surgical decision-making when age is the primary barrier to intervention.

## Introduction

Rheumatoid arthritis (RA) is a chronic systemic autoimmune disease characterized by persistent synovial inflammation, progressive cartilage destruction, and osseous erosion, affecting approximately 0.5%-1% of adults worldwide [1]. Early initiation of disease-modifying antirheumatic drugs (DMARDs) during the therapeutic “window of opportunity” has been shown to reduce radiographic progression and improve long-term functional outcomes. Even delays of several months in treatment initiation have been associated with irreversible structural damage that may not be fully mitigated by later escalation of therapy [2].

Shoulder involvement is common in RA, with symptoms reported in up to 65%-90% of patients and radiographic abnormalities developing bilaterally in nearly half of patients within five years of disease onset [3-4]. Although progression to end-stage glenohumeral arthropathy typically occurs later in life, severe destructive disease may develop in younger individuals, particularly in the setting of aggressive inflammatory activity or delays in optimized medical management.

Reverse total shoulder arthroplasty (RTSA) reverses the native ball-and-socket configuration by placing a glenosphere on the glenoid and a concave component on the humerus. Unlike anatomic total shoulder arthroplasty, which depends on a functional rotator cuff to center and stabilize the humeral head, RTSA medializes the center of rotation and distalizes the humerus, increasing the deltoid’s tension and mechanical advantage and allowing active elevation despite rotator cuff deficiency, consistent with the biomechanical principles described by Grammont [5]. 

Although RTSA has demonstrated favorable outcomes in RA and other cuff-deficient conditions, most published cohorts involve older adults, leaving limited evidence regarding long-term implant durability in very young patients [6-9]. This uncertainty, together with the potential lifetime revision burden, contributes to continued hesitancy when considering RTSA in patients younger than 40 years [10].

In the present case, the patient initially sought evaluation from a shoulder specialist who declined arthroplasty based primarily on age and anticipated revision risk. Such caution reflects broader uncertainty in the management of very young patients with inflammatory shoulder destruction. We report RTSA performed in a 25-year-old woman with seropositive RA who achieved excellent two-year functional outcomes. By contextualizing these results within the existing literature on inflammatory and younger RTSA cohorts, this report contributes to the evolving discussion regarding surgical decision-making in carefully selected young patients with end-stage inflammatory shoulder disease.

## Case presentation

A 25-year-old woman with seropositive RA presented with progressive right shoulder pain, stiffness, and functional limitation. She developed migratory inflammatory polyarthritis involving the hands, wrists, feet, and shoulders in October 2020 and was formally diagnosed with RA in December 2020. Initial testing showed a rheumatoid factor of 89 IU/mL, an anti-CCP antibody level of 30.8, an ESR of 56 mm/h, and a CRP of 31.3 mg/L; Disease Activity Score 28 (DAS28) scores were not available. Hydroxychloroquine was initiated at diagnosis, followed by methotrexate in April 2021 after a five- to six-month delay related to medication hesitancy and limited in-person access during the COVID-19 pandemic. Adalimumab was initiated in 2022 for persistent disease activity and later changed to etanercept because of recurrent flares. Hydroxychloroquine was discontinued in September 2022 because of a possible adverse effect.

Right shoulder involvement was documented by January 2022, with worsening pain and approximately one hour of morning stiffness. By December 2022, she had persistent pain, limited motion, synovitis, and reportedly advanced radiographic degeneration. To delay surgery, she completed 14 physical therapy sessions from December 2022 through July 2023, with an approximately three-month interruption. Range of motion, strengthening, stabilization, manual therapy, and home exercises provided some improvement, but pain persisted at up to 8/10, with crepitus and difficulty reaching, lifting, and elevating the arm. An early flare was treated with an oral methylprednisolone taper, and right glenohumeral methylprednisolone acetate injections were performed in December 2022 and April 2023. Despite systemic therapy and these interventions, her symptoms and mechanical dysfunction continued to progress. By September 2023, approximately 20 months after documented shoulder involvement, she had developed end-stage destructive arthropathy, although the precise timing of structural collapse could not be determined without serial imaging.

Despite systemic disease management, she developed progressively worsening mechanical symptoms localized to the right shoulder that were refractory to conservative measures. Preoperative examination demonstrated marked functional impairment, including pseudoparalysis with active forward elevation of 60°, passive forward elevation of 90°, external rotation of 40°, and internal rotation to L5. Supraspinatus strength was 0/5, with additional infraspinatus and subscapularis weakness. Palpable glenohumeral crepitus and an inability to lift the arm accompanied pain that substantially limited activities of daily living, recreation, and quality of life, consistent with advanced inflammatory arthropathy.

Magnetic resonance imaging and radiographs obtained in September 2023 demonstrated advanced destructive glenohumeral arthropathy, including complete joint space obliteration, humeral head flattening with erosive changes and central avascular necrosis with an unstable fractured fragment, subchondral cyst formation, a full-thickness retracted supraspinatus tear, substantial infraspinatus tearing, gross synovitis, and a large joint effusion. These findings were consistent with end-stage inflammatory shoulder destruction and rotator cuff insufficiency (Figures 1, 2).

**Figure 1:**
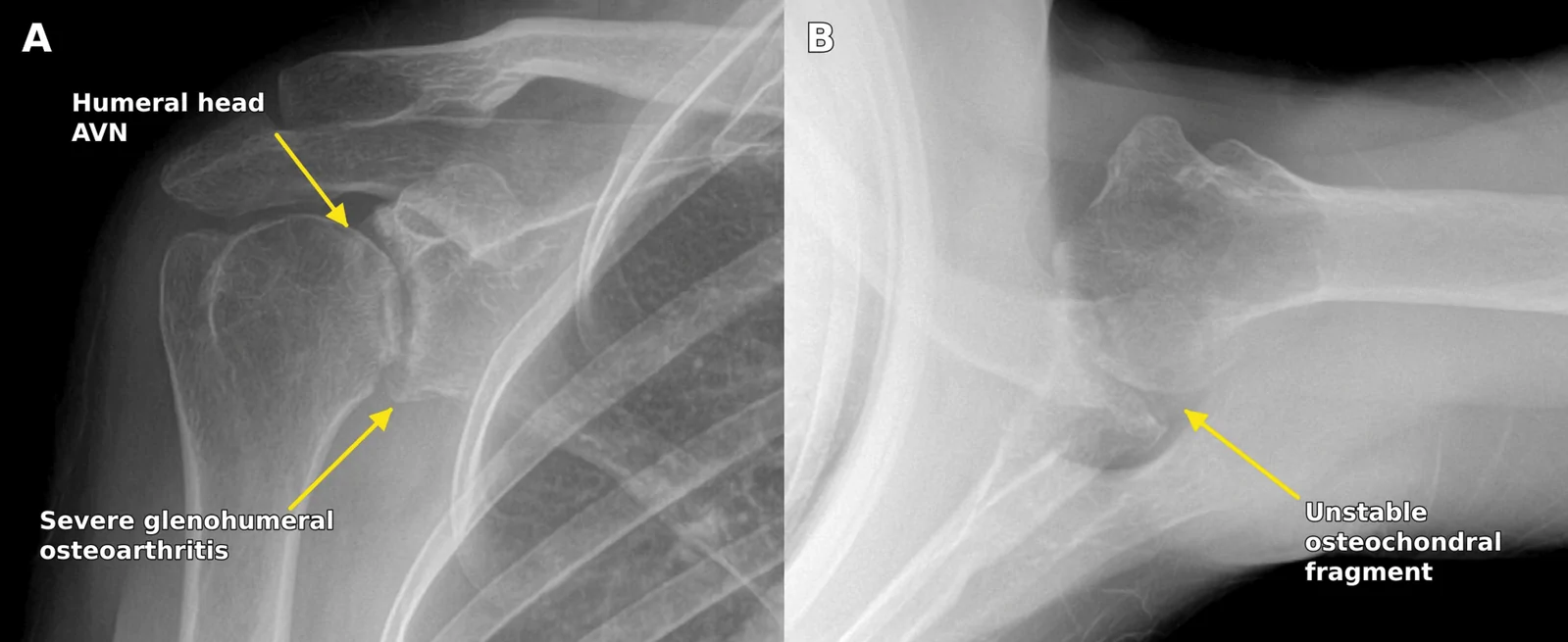
Presenting radiographs of the right shoulder. (A) Anteroposterior view demonstrating humeral head avascular necrosis (AVN; upper arrow) and severe glenohumeral osteoarthritis (lower arrow). (B) Axillary lateral view demonstrating an unstable osteochondral fragment (arrow).

**Figure 2:**
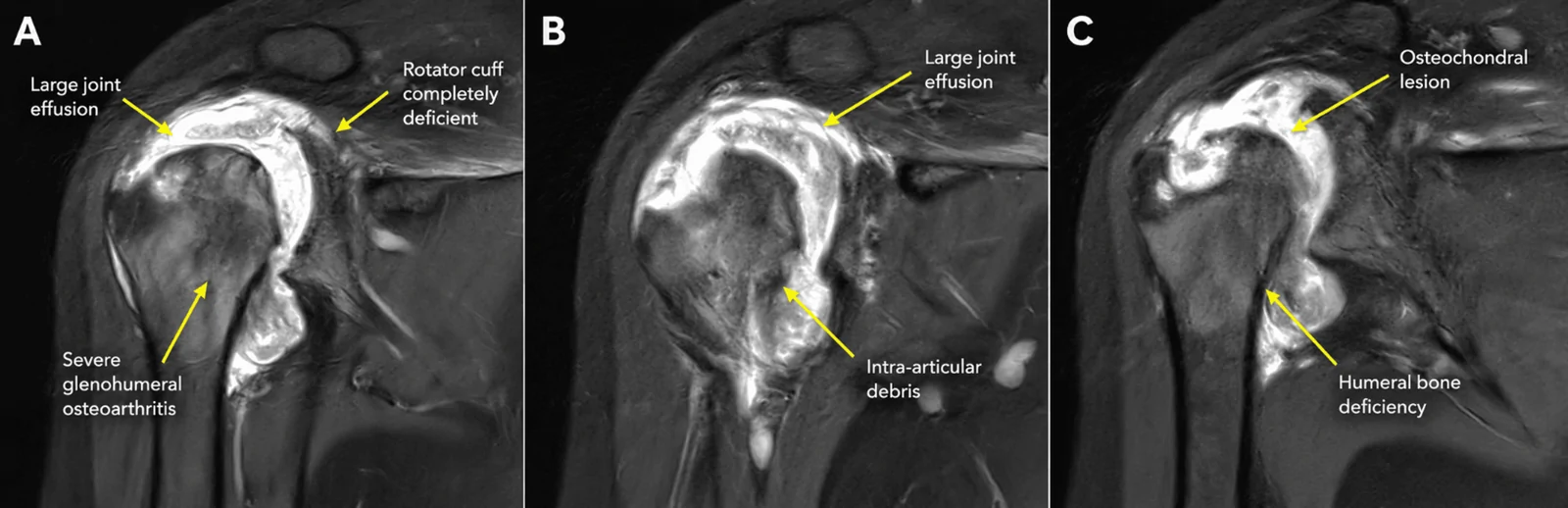
Preoperative magnetic resonance imaging of the right shoulder demonstrating advanced destructive inflammatory arthropathy. (A) Coronal proton density (PD) image demonstrating a large glenohumeral joint effusion, severe glenohumeral degenerative changes, and rotator cuff insufficiency. (B) Coronal PD image demonstrating a large joint effusion containing intra-articular debris. (C) Coronal PD image demonstrating an osteochondral lesion with humeral head bone loss and structural deficiency.

Orthopedic evaluation documented more than five months of progressive shoulder pain despite supervised physical therapy and optimized medical management. She had received multiple courses of systemic corticosteroids, including oral methylprednisolone tapers for inflammatory flares, without durable improvement. There was no evidence of medication nonadherence following initiation of rheumatologic therapy. She denied prior shoulder trauma, instability, or history of inflammatory arthritis during childhood or adolescence.

In October 2023, fluoroscopy-guided aspiration of the right glenohumeral joint was performed because of the large effusion and concern for infection. A Gram stain showed rare white blood cells and no organisms. Routine fluid culture showed no growth, and anaerobic culture showed no anaerobes isolated. A quantitative synovial-fluid cell count, differential, and crystal-analysis result were not available.

Although the preoperative aspiration was negative, the marked effusion, gross synovitis, and unusually advanced joint destruction in this young patient warranted a staged approach to further exclude occult infection before prosthetic implantation. Unlike a non-staged primary RTSA, in which debridement and definitive implant placement occur during a single operation, this approach separated diagnostic and therapeutic arthroscopy from definitive reconstruction. In November 2023, the patient underwent right shoulder diagnostic arthroscopy with extensive synovectomy, tissue biopsies for culture, loose body removal, glenohumeral chondroplasty, labral and rotator cuff debridement, and subacromial decompression. This procedure allowed direct assessment of the articular damage, rotator cuff deficiency, and bone loss while addressing the intra-articular pathology. Intraoperative findings confirmed advanced destructive inflammatory arthropathy, and all cultures were negative (Figure 3).

**Figure 3:**
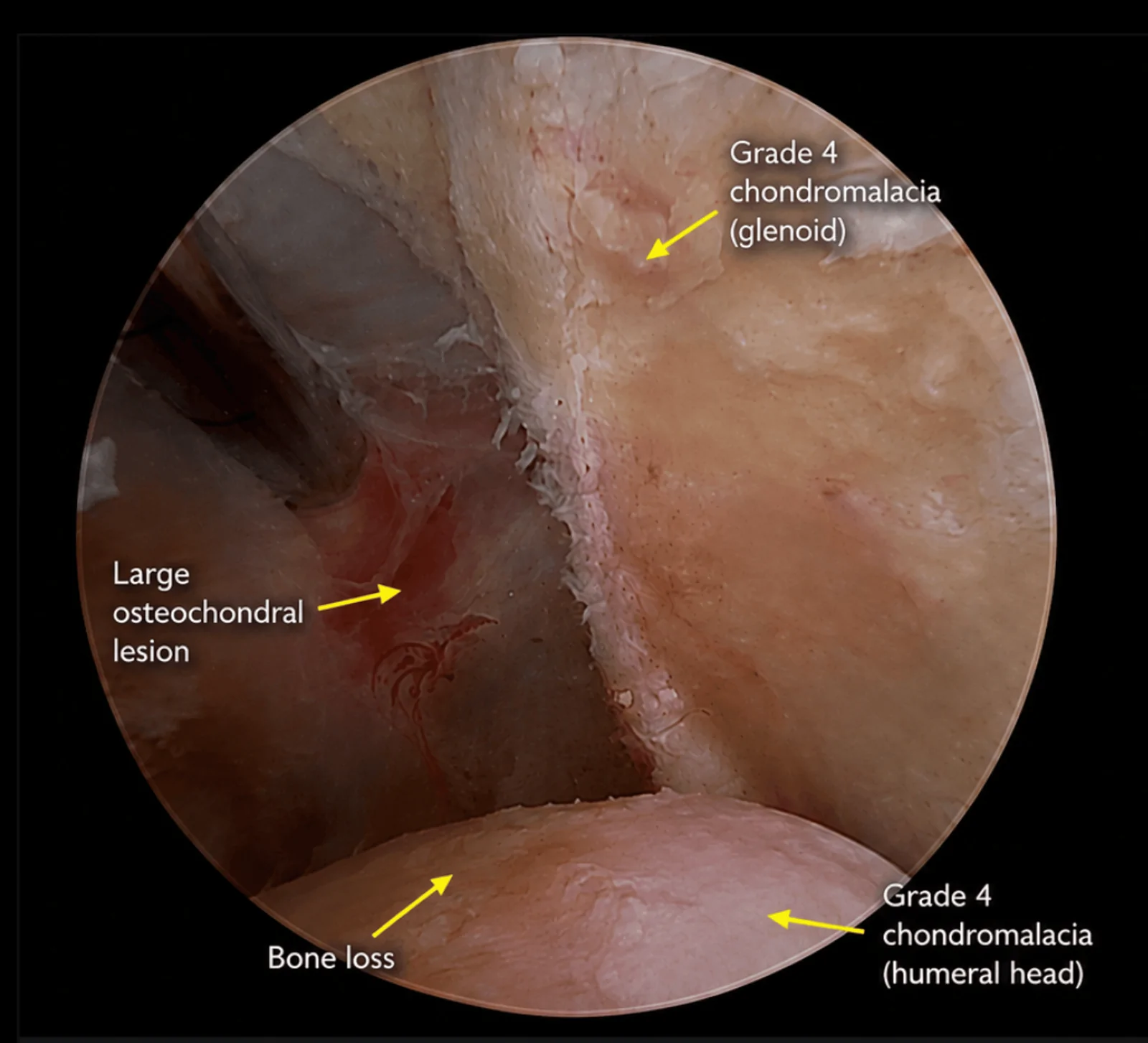
Diagnostic arthroscopy of the right shoulder demonstrating a large osteochondral lesion with humeral head bone loss and grade IV chondromalacia of the glenoid and humeral head, characterized by full-thickness cartilage loss with exposed subchondral bone.

Given the combination of end-stage glenohumeral destruction and irreparable rotator cuff insufficiency, RTSA was selected over anatomic total shoulder arthroplasty. Evidence supporting its ability to improve pain and function in patients with RA and other cuff-deficient conditions further supported this choice [8,11]. Definitive RTSA was performed in December 2023 without intraoperative or early postoperative complications (Figure 4).

**Figure 4:**
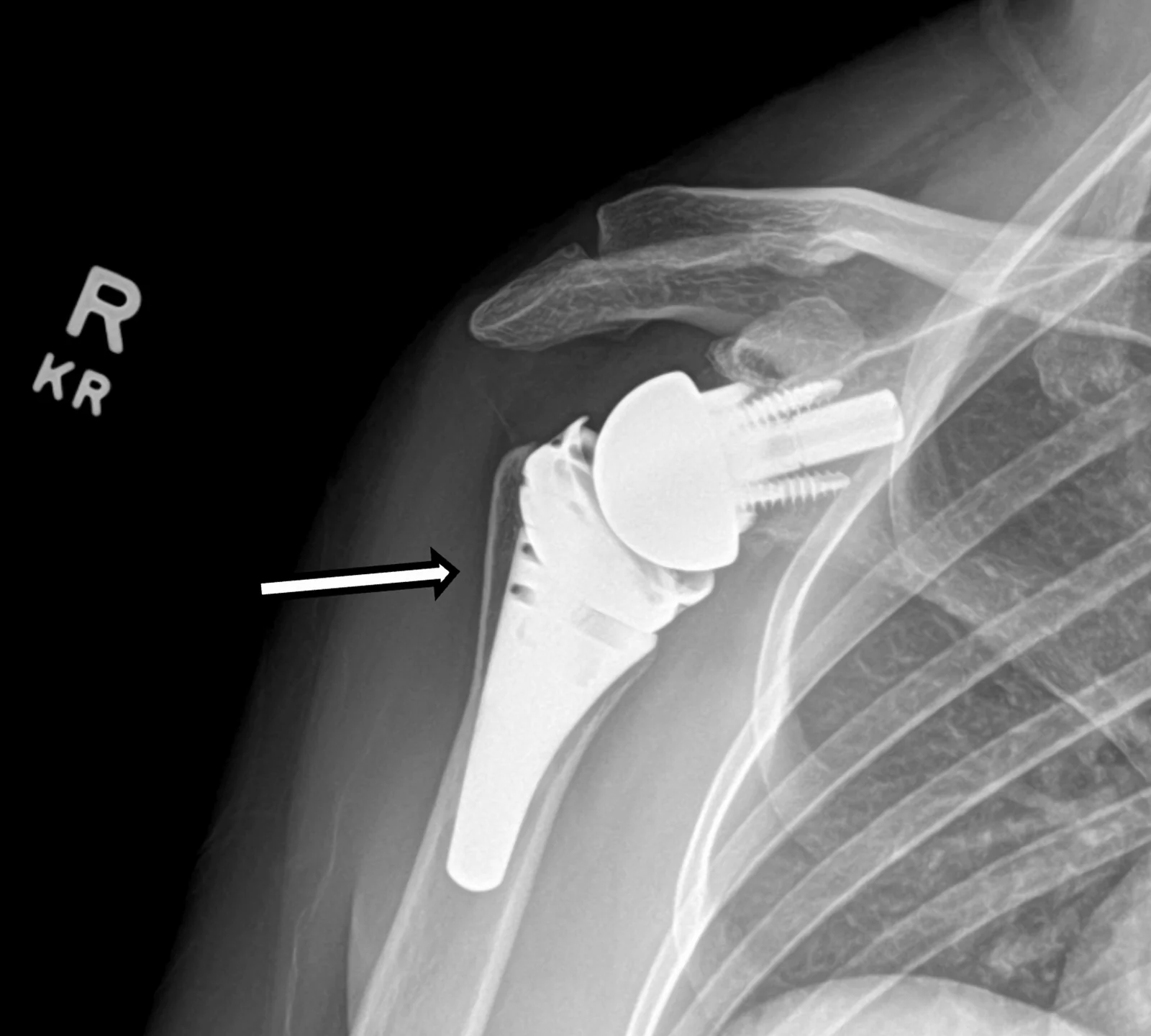
Postoperative X-ray demonstrating reverse total shoulder arthroplasty.

Perioperative medication management was coordinated with rheumatology. Weekly methotrexate and etanercept were withheld for approximately one week before and after surgery, consistent with the preoperative orthopedic instructions to skip the upcoming doses. She was not receiving chronic systemic corticosteroid therapy and had no substantial prednisone requirement at the time of surgery.

At two weeks postoperatively, the incision was clean and dry without signs of infection. Following six weeks of sling immobilization, the patient began supervised physical therapy focused on progressive range of motion and strengthening. At 15 weeks, active flexion was 165°, abduction 155°, and external rotation 55°, with pain rated 0-1/10. By four months, she had regained near-full active range of motion, with only mild limitation in terminal internal rotation, and reported high satisfaction with her recovery. After completing three months of supervised therapy, she transitioned to a structured home exercise program.

At six months, she had full, painless range of motion, improved strength, normal shoulder stability, and no need for analgesic medication, although she occasionally experienced mild discomfort with certain movements. At one year, she maintained full, painless range of motion with 5/5 rotator cuff strength. Serial radiographs obtained at two weeks, six months, and one year demonstrated an intact implant without loosening or mechanical failure. Throughout follow-up, there were no episodes of infection or instability.

At two years, she remained independent in activities of daily living, including overhead reaching, grooming, and work-related tasks, with only mild difficulty sleeping on the operative side. Patient-reported outcomes were assessed using the American Shoulder and Elbow Surgeons (ASES) Standardized Shoulder Assessment Form, a public-domain instrument of the ASES, and the freely available single-item Single Assessment Numeric Evaluation (SANE) measure [12,13]. She achieved an ASES score of 80/100, reflecting mild pain (3/10) and high functional capacity (Activities of Daily Living score, 27/30), and a SANE score of 95%, indicating excellent perceived shoulder function. Formal preoperative ASES and SANE scores were not collected, precluding calculation of within-patient changes. The patient's clinical timeline of treatment, shoulder disease progression, surgical management, and outcomes are summarized in Table 1.

**Table 1:** Clinical timeline of treatment, shoulder disease progression, surgical management, and outcomes Formal preoperative ASES and SANE scores were not collected; therefore, within-patient changes in these measures could not be calculated. Abbreviations: ASES, American Shoulder and Elbow Surgeons; anti-CCP, anti-cyclic citrullinated peptide; CRP, C-reactive protein; ESR, erythrocyte sedimentation rate; RA, rheumatoid arthritis; RTSA, reverse total shoulder arthroplasty; SANE, Single Assessment Numeric Evaluation.

Date or time point	Clinical course and outcomes
October–December 2020	Migratory inflammatory polyarthritis developed. Seropositive RA was diagnosed in December 2020, and hydroxychloroquine was initiated.
April 2021	Methotrexate was initiated approximately five to six months after symptom onset.
January–September 2022	Right shoulder pain and morning stiffness were documented. Adalimumab was initiated for persistent disease activity and later changed to etanercept because of recurrent flares. Hydroxychloroquine was discontinued in September 2022 because of a possible adverse effect.
December 2022–April 2023	Persistent shoulder pain, synovitis, limited motion, and advanced radiographic degeneration were documented. Oral methylprednisolone tapers and glenohumeral corticosteroid injections in December 2022 and April 2023 provided no durable improvement.
September 2023	Examination showed pseudoparalysis, active forward elevation of 60°, and supraspinatus strength of 0/5. Radiographs and MRI demonstrated end-stage glenohumeral destruction, humeral head collapse and avascular necrosis, a large effusion, and extensive rotator cuff tearing.
October 2023	Glenohumeral aspiration showed no organisms, and routine and anaerobic cultures were negative.
November 2023	Diagnostic arthroscopy with synovectomy, tissue biopsy, loose-body removal, chondroplasty, and debridement confirmed advanced cartilage loss, bone loss, and rotator cuff deficiency. All intraoperative cultures were negative.
December 2023	RTSA was performed without complication. Methotrexate and etanercept were withheld for approximately one week before and after surgery.
2 weeks–3 months post operation	The incision was clean and dry at two weeks. After six weeks of sling immobilization, supervised physical therapy was completed for three months, followed by a home exercise program.
15 weeks–4 months post operation	Active flexion was 165°, abduction 155°, and external rotation 55°, with pain rated 0–1/10. Near-full active motion and high satisfaction were reported by four months.
6 months post-operation	The patient had full, painless motion, improved strength, normal stability, and no analgesic requirement. Radiographs showed an intact implant without loosening or mechanical failure.
1 year post operation	Full, painless motion and 5/5 strength were maintained. Radiographs demonstrated an intact implant without loosening or mechanical failure.
2 years post operation	The patient remained independent in daily activities. The ASES score was 80/100, including pain of 3/10 and an activities of daily living score of 27/30, and the SANE score was 95%. No infection, instability, or mechanical failure was identified during follow-up.

## Discussion

This case illustrates aggressive, predominantly monoarticular shoulder destruction in a very young patient with seropositive RA. Although RA most commonly necessitates shoulder arthroplasty later in life, this patient developed rapid structural collapse at 25 years of age despite systemic therapy. The presence of humeral head avascular necrosis, advanced erosive change, and irreparable rotator cuff insufficiency eliminated joint-preserving or anatomic arthroplasty options.

Most published RTSA outcomes in RA involve substantially older patients. A systematic review of 123 shoulders reported improvement in mean ASES scores from 27.5 to 73.7 and a reduction in mean Visual Analog Scale (VAS) pain scores from 7.3 to 1 [6]. Medium-term data demonstrated approximately 96% revision-free survivorship at seven years and improvement in active anterior elevation from approximately 65° to greater than 130° [7]. Gee et al. reported an overall complication rate of 19%, including fractures (10.7%), infection or septic loosening (3.3%), symptomatic glenoid loosening (1.7%), nerve palsy (1.7%), and dislocation (0.8%). Scapular notching occurred in 42% of evaluated shoulders, and revision was required in 7% overall [8]. Although these findings support the effectiveness of RTSA in RA, their applicability to a 25-year-old with decades of anticipated implant use remains uncertain.

The medical record documented that arthroplasty was initially deferred because of the patient’s age and anticipated lifetime revision burden. RTSA was ultimately selected because of end-stage joint destruction, irreparable rotator cuff insufficiency, instability, failure of nonoperative treatment, and a negative infection workup. Anatomic total shoulder arthroplasty was unsuitable because it relies on a functional rotator cuff, while hemiarthroplasty may result in progressive glenoid wear. Factors warranting caution in very young patients include active infection, deltoid or axillary nerve dysfunction, inadequate glenoid bone stock, poor bone quality, immunosuppression-related risks, and anticipated lifetime revision burden.

In younger populations, evidence remains limited and outcomes are more heterogeneous. An analysis of the New Zealand Joint Registry (2000-2019) identified 5,518 primary RTSAs, of which only 75 patients (1.4%) were younger than 55 years (range, 34-54 years). Inflammatory arthritis accounted for 17% of indications in this younger cohort, with a mean follow-up of 2.36 years [9]. A systematic review of RTSA in patients younger than 65 years (mean age, 57 years; 245 patients) reported a weighted mean ASES score of 67, an overall complication rate of 18%, and a reoperation rate of 6% at approximately 34 months [14]. Although this patient’s ASES score of 80 at two years was numerically higher, direct comparison is limited by differences in age, indications, baseline pathology, study design, and follow-up. Younger patients are also less likely to achieve clinically significant improvements in SANE scores than older patients, making her postoperative SANE score of 95% noteworthy [15]. Longer-term studies demonstrate sustained improvement and high implant survivorship at up to 12 years, although complication profiles may differ from those of older cohorts [10]. Against this background, the patient’s near-full elevation and favorable patient-reported outcomes are encouraging and may have been supported by continued DMARD adherence, an uncomplicated postoperative course, and engagement with rehabilitation.

Published reports of RTSA in patients younger than 30 years with RA remain especially sparse. However, the two-year follow-up remains early relative to this patient’s expected implant lifespan, and the findings of a single case may not be generalizable. Annual clinical and radiographic follow-up is planned to monitor function, component position, loosening, osteolysis, scapular notching, and instability, with revision considered for symptomatic or progressive component loosening or migration, recurrent instability, periprosthetic fracture, infection, or substantial functional decline. Despite these long-term uncertainties, age alone should not be considered an absolute contraindication when joint destruction is advanced, and nonoperative management has failed. Instead, candidacy should account for disease severity, functional impairment, rotator cuff integrity, and patient goals, together with thorough exclusion of infection, realistic counseling regarding implant longevity, and careful surgical planning. In appropriately selected young patients, RTSA may restore meaningful function and quality of life.

## Conclusions

RTSA may provide substantial pain relief and restore active elevation and function in carefully selected young patients with end-stage inflammatory shoulder disease and irreparable rotator cuff insufficiency. The favorable two-year outcome in this patient is encouraging; however, a single case cannot establish long-term implant durability or predict outcomes in all young patients with RA. When nonoperative management has failed, candidacy for RTSA should be individualized according to joint destruction, rotator cuff integrity, functional impairment, bone stock, infection status, and patient goals rather than chronological age alone.
